# Fetal Brain Biometric Measurements on 3D Super-Resolution Reconstructed T2-Weighted MRI: An Intra- and Inter-observer Agreement Study

**DOI:** 10.3389/fped.2021.639746

**Published:** 2021-08-10

**Authors:** Marie Khawam, Priscille de Dumast, Pierre Deman, Hamza Kebiri, Thomas Yu, Sébastien Tourbier, Hélène Lajous, Patric Hagmann, Philippe Maeder, Jean-Philippe Thiran, Reto Meuli, Vincent Dunet, Meritxell Bach Cuadra, Mériam Koob

**Affiliations:** ^1^Department of Radiology, Lausanne University Hospital, University of Lausanne (CHUV-UNIL), Lausanne, Switzerland; ^2^CIBM Center for Biomedical Imaging, Lausanne, Switzerland; ^3^Signal Processing Laboratory (LTS5), Ecole Polytechnique Fédérale de Lausanne, Lausanne, Switzerland

**Keywords:** fetal, brain, biometry, magnetic resonance imaging, sequences, super-resolution

## Abstract

We present the comparison of two-dimensional (2D) fetal brain biometry on magnetic resonance (MR) images using orthogonal 2D T2-weighted sequences (T2WSs) vs. one 3D super-resolution (SR) reconstructed volume and evaluation of the level of confidence and concordance between an experienced pediatric radiologist (obs1) and a junior radiologist (obs2). Twenty-five normal fetal brain MRI scans (18–34 weeks of gestation) including orthogonal 3-mm-thick T2WSs were analyzed retrospectively. One 3D SR volume was reconstructed per subject based on multiple series of T2WSs. The two observers performed 11 2D biometric measurements (specifying their level of confidence) on T2WS and SR volumes. Measurements were compared using the paired Wilcoxon rank sum test between observers for each dataset (T2WS and SR) and between T2WS and SR for each observer. Bland–Altman plots were used to assess the agreement between each pair of measurements. Measurements were made with low confidence in three subjects by obs1 and in 11 subjects by obs2 (mostly concerning the length of the corpus callosum on T2WS). Inter-rater intra-dataset comparisons showed no significant difference (*p* > 0.05), except for brain axial biparietal diameter (BIP) on T2WS and for brain and skull coronal BIP and coronal transverse cerebellar diameter (DTC) on SR. None of them remained significant after correction for multiple comparisons. Inter-dataset intra-rater comparisons showed statistical differences in brain axial and coronal BIP for both observers, skull coronal BIP for obs1, and axial and coronal DTC for obs2. After correction for multiple comparisons, only axial brain BIP remained significantly different, but differences were small (2.95 ± 1.73 mm). SR allows similar fetal brain biometry as compared to using the conventional T2WS while improving the level of confidence in the measurements and using a single reconstructed volume.

## Introduction

Biometric measurements are good markers of fetal brain maturation and growth and are a fundamental basis for the diagnosis of developmental and acquired brain abnormalities (1). Indeed, an abnormal measurement is often the first warning of disturbed fetal growth that requires further investigation. Quantifying brain development, in comparison to reference charts, is the first routine step of prenatal diagnosis on ultrasound (US) and magnetic resonance imaging (MRI). This can detect common pathologies like microcephaly, cerebellar hypoplasia, corpus callosum dysgenesis, and ventriculomegaly (2). The accuracy of biometric data is essential for the evaluation of prognosis and prediction of outcome as it may influence both prenatal and postnatal management. Indeed, it is crucial to give appropriate parental information and counseling, as termination of pregnancy may be considered in severe cases (3).

US is the first screening fetal brain imaging modality. MRI comes as a complementary imaging tool to confirm or rule out pathological US findings and to look for additional information (2). Fetal biometry performed on US may be influenced by maternal morphology, fetal position, or interference from the skull. MRI does not share these limitations and, in contrast to US, provides more accurate measurements of the fetal brain, together with better parenchymal signal and gyration analysis. Moreover, although some studies have demonstrated a good concordance between both techniques for biometry (4–6), others have shown discrepancies (7). For example, MRI has been found to be more accurate for the definitive diagnosis of microcephaly (8, 9), as preliminary US diagnoses were ruled out in 8 out of 30 cases after subsequent MRI analysis. This is valuable, as a small brain size is considered a risk of poor neurodevelopmental outcome. Similar conclusions were drawn from studies on the corpus callosum or the vermis (10, 11).

In clinical practice, fetal brain MRI biometry is performed on series of two-dimensional (2D) 3- to 4-mm-thick slices acquired in three orthogonal planes by T2-weighted sequences (T2WSs). These T2WS-based measurements are then compared to reference values (4, 12–15). In two large-scale studies, a good inter-rater agreement on T2WS was found for most brain biometric measurements (4, 16). However, a key limitation of fetal MRI is the maternal and fetal motion, which often results in inter-slice and inter-series motion as well as intensity artifacts. The acquisition plane may be oblique, thus not accurately oriented in the reference orthogonal planes, and motion may occur between acquisition planes, which makes identification of anatomical landmarks difficult and hampers subsequent measurement. Therefore, fetal brain MRI biometric analysis based on T2WS is commonly performed on low-quality images with low confidence and uncertainty. The level of confidence is another important parameter in the process of image analysis (5), as it corresponds to the “degree of certainty in the correctness of diagnosis” and is associated with relevant decisional consequences (17, 18). An imaging method where measurements would be easier to perform could help in providing accurate biometry with more confidence, particularly on suboptimal images and for less experienced radiologists.

**Figure 1:**
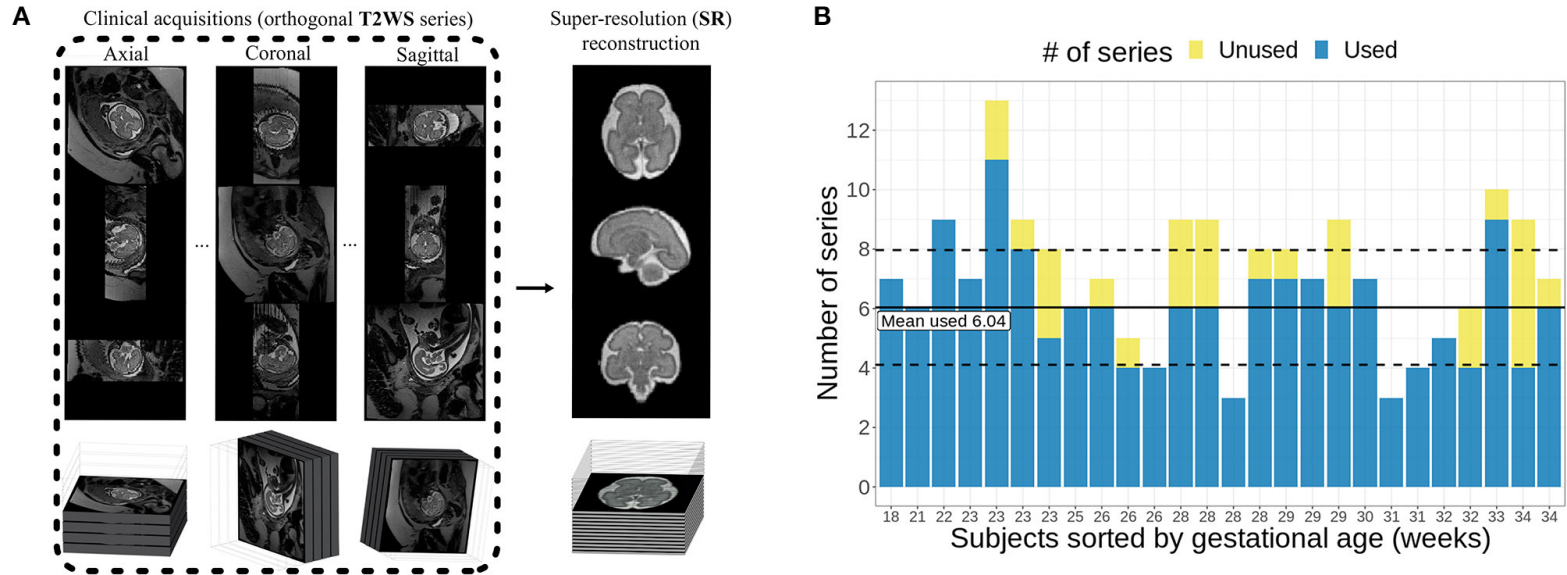
**(A)** Illustration of primary T2-weighted Half-Fourier Acquisition Single-shot Turbo spin Echo (HASTE) images and the motion-free super-resolution (SR) reconstructed volume (Figure adapted from (32)). **(B)** Distribution of the number of series used per subject compared to the total available number of series.

The recent developments of advanced image processing methods based on super-resolution (SR) techniques allow the reconstruction of a 3D high-resolution motion- and inhomogeneity-free volume from T2WSs (19–26). Handling a 3D volume with isotropic spatial resolution could be of great value to conduct fetal brain biometry, as it would allow to freely navigate in any plane and to obtain SR orthogonal planes easily (27–30), as well as automated quantitative studies (31) of high-resolution 3D images. As depicted in Figure 1A, fetal brain SR reconstruction aims at combining orthogonal low-resolution series of thick 2D slices as acquired clinically into a single isotropic high-resolution image free of motion artifacts (the SR-reconstructed volume) that enables the visualization of fetal brain anatomy in any plane.

This study aimed at comparing 2D measurements of fetal brain biometry using three orthogonal 2D low-resolution T2WSs vs. using one single 3D SR-reconstructed volume for both supratentorial and infratentorial measurements. Three points were evaluated: the agreement between T2WS and SR measurements, the level of the observers' confidence on both datasets, and the concordance between a junior radiologist and an experienced pediatric radiologist.

## Materials and Methods

### Dataset

#### Cohort

We retrospectively collected all consecutive normal fetal brain MRI exams from January 2013 to October 2018 (28 subjects in total: 15 males and 13 females) from the MRI database of our institution. All MRI scans were conducted on medical indication (ventriculomegaly, suspicion of corpus callosum or posterior fossa anomaly, microcephaly…) within 2 weeks of an expert fetal neurosonographic study and were finally considered normal. Early neonatal clinical evaluation was normal. All images were anonymized prior to further analysis. This retrospective study was part of a larger research protocol at our institution approved by the local ethics committee.

#### MRI

Clinical MR images were acquired either at 1.5 T (MAGNETOM Aera, Siemens Healthcare, Erlangen, Germany) (88% of the subjects) or at 3 T (MAGNETOM Skyra^fit^, Siemens Healthcare, Erlangen, Germany) (12% of the subjects). The fetal brain MRI protocol included T2-weighted (T2W) Half-Fourier Acquisition Single-shot Turbo spin Echo (HASTE) sequences in the three orthogonal orientations; usually at least two acquisitions were performed in each orientation, together with axial gradient echo T1-weighted and diffusion-weighted imaging (DWI) or diffusion tensor imaging (DTI) in some cases. The coronal plane was parallel to the brain stem, and the axial plane was parallel to the corpus callosum long axis (4). We excluded twins from the study (*n* = 2). At this point, 26 normal fetal brain MR images were kept for further analysis. Details on the MRI acquisition parameters can be found in Table 1.

**Table 1:** MRI acquisition parameters of the T2-weighted Half-Fourier Acquisition Single-shot Turbo spin Echo (HASTE) sequences.

**Field strength (Tesla)**	**Number of exams**	**Gestational age (weeks)**	**Number of series**	**In-plane resolution** **(mm)**		**Slice thickness** **(mm)**		**Echo time** **(ms)**		**Repetition time** **(ms)**	
				**Min–Max**	**Mean ± SD**	**Min–max**	**Mean ± SD**	**Min–Max**	**Mean ± SD**	**Min–Max**	**Mean ± SD**
1.5 T	23	18–3427.4 ± 4.2	143	1.125–1.172	1.127 ± 0.009	2.42–4.0	3.34 ± 0.20	82–98	90 ± 1.9	832–1,200	1,185 ± 59
3 T	3		14	0.547	–	3.0	–	101	–	1,000–1,100	1,090 ± 027

### Methodology

#### Three-Dimensional Super-Resolution Reconstruction

In our study, 3D SR volumes were reconstructed from T2WSs within the PACS station with an in-house *syngo*.via Frontier fetal MRI prototype (33) based on the publicly available MIALSRtoolkit software (20, 34). In a nutshell, the fetal brain MRI prototype performs the following steps: fetal brain extraction, bias field correction, inter-slice motion estimation based on slice-to-volume rigid registration, and SR reconstruction based on a forward model of the image acquisition process that leads to an inverse problem that is solved *via* an efficient total variation algorithm with automatic regularization (20).

All cases were reconstructed by an engineer with 20 years of experience in medical image processing. The selection of the series used for the reconstruction was done based on visual inspection, and T2WSs that exhibited high levels of motion distortion and/or intensity signal dropout (thus, that were not exploitable for radiological reading neither) were excluded from the 3D SR reconstruction process. On average, six series were used per subject for SR reconstruction, with a range from 3 to 11 series (Figure 1B). All 3D images were reconstructed with an isotropic spatial resolution matching its input in-plane resolution (in average of around 1.1 × 1.1 × 1.1 mm^3^).

#### Evaluation of Super-Resolution Quality

**Figure 2:**
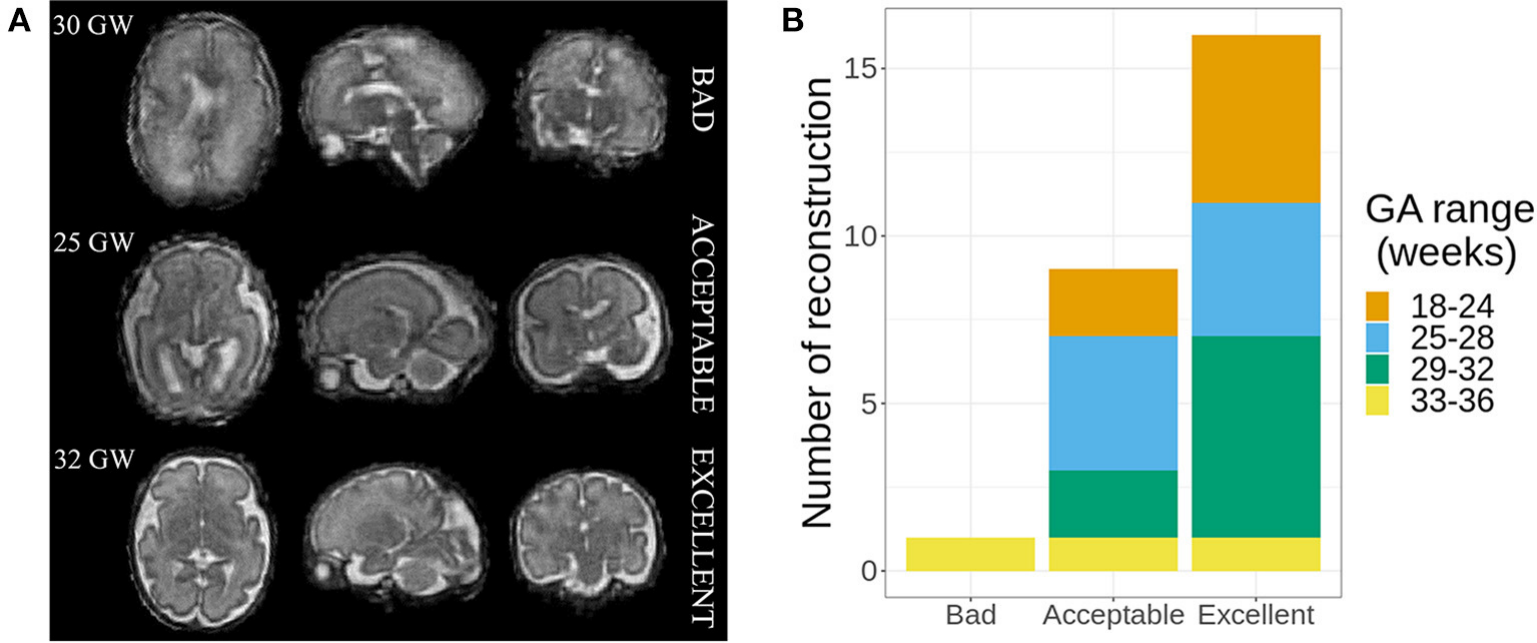
**(A)** Examples of the three different ratings made by the experts. **(B)** Quality of super-resolution (SR) reconstructions, with the number of cases in each category of gestational age (GA) range.

The quality of the 3D SR was assessed independently, in a blinded protocol, by three expert raters: one engineer expert in MR image analysis (rater 1, the same who performed the SR reconstructions) and two experienced pediatric radiologists (rater 2 and rater 3, with, respectively, 15 and 9 years of experience in fetal brain MRI). Reconstructed volumes were classified into one of three categories: bad (with remaining motion, very blurred, unusable for diagnosis purposes), acceptable (overall good quality with some blurring but still relevant for diagnosis purposes), or excellent (good quality without any blurring). Examples of SR-reconstructed volumes rated with the three different quality measures are shown in Figure 2A.

#### Biometric Measurements

Using standard tools on the PACS station (Carestream Vue PACS®, Version 12.1.6.1005, Carestream Health Inc., NY, USA), two observers, one experienced pediatric neuroradiologist (obs1, 15 years of experience of fetal brain MRI) and one inexperienced radiologist (obs2, without any experience in fetal brain MRI) independently measured 11 fetal brain structures on both datasets (T2WS then SR) during two reading sessions separated by 3 weeks and blinded to the results of the previous measurements.

**Figure 3:**
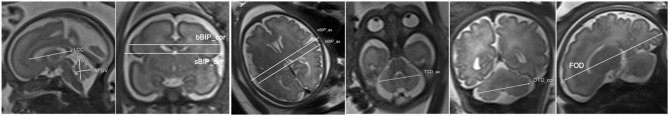
Biometric measurements. LCC, length of the corpus callosum; APDV, anteroposterior diameter of the vermis; HV, height of the vermis; bBIP_cor, sBIP_cor, bBIP_ax, sBIP_ax, brain and skull biparietal diameter (coronal and axial); TCD_ax and TCD_cor, transverse cerebellar diameter (axial and coronal); FOD, fronto-occipital diameter.

The following biometric measurements as shown in Figure 3 were performed following previously published guidelines for fetal brain MRI biometry (2, 4, 12, 28).

The skull coronal biparietal diameter (sBIP_cor) is defined as the greatest transversal diameter between the inner tables of parietal bones on a coronal slice through the temporal horns of the lateral ventricles. The brain coronal biparietal diameter (bBIP_cor) was measured on the same slice.

The brain axial biparietal diameter (bBIP_ax) is defined as the maximal brain diameter in the transverse plane through the atria. The skull axial biparietal diameter (sBIP_ax) is defined as the inner to inner table maximal skull diameter in the transverse plane through the atria.

The corpus callosum length (LCC), height of the vermis (HV), and anteroposterior vermis diameter (APDV) were measured in the mid-sagittal plane.

The transverse cerebellar diameter was measured on a coronal slice (TCD_cor) and on an axial slice (TCD_ax).

The fronto-occipital diameters [right fronto-occipital diameter (rFOD) and left fronto-occipital diameter (lFOD)] were measured on a sagittal slice between the extreme points of the frontal and occipital cortices.

On T2WS, each observer independently chose the best-quality T2W series for each measurement. SR measurements were performed in orthogonal planes in multiplanar reformations (MPRs) using the tools within the PACS system. Each observer rated the confidence of each of his measurements in both T2WS and SR datasets either as high or low.

#### Statistical Analysis

Statistical analysis was conducted with R software (version 3.6.3).

##### Evaluation of Super-Resolution Quality

The inter-rater reliability was measured using a weighted ordinal Gwet's agreement coefficient (Gwet's AC) and interpreted according to Altman's benchmarking scale (35).

##### Level of Confidence

Chi-square test statistics were used to evaluate the dependence of the level of confidence of the raters on each dataset.

##### Biometric Measurements

The association of paired lFOD and rFOD measurements was tested using Spearman's *rho* statistic (36), and the difference was tested with the paired Wilcoxon rank sum test. This analysis was performed for each pair of dataset–observer. Biometric measurements were compared statistically to determine inter-dataset and inter-observer significant difference, respectively, for each observer (obs1 and obs2) or dataset (T2WS and SR), with the paired Wilcoxon rank sum test (without and with Bonferroni multiple comparisons correction). Lin's concordance correlation coefficient (CCC) (37, 38) and intraclass correlation coefficient (ICC) were computed for the agreement on measurements obtained on the two datasets (39). Bland–Altman plots were used to assess the agreement between the two observers for each of the two datasets and the reliability between the two datasets for each of the two observers. Agreement was rated as follows: poor, <0.5; moderate, 0.5–0.75; good, 0.75–0.9; excellent, >0.90 (35). Agreement between transverse and coronal measurements was evaluated for brain (bBIP_ax, bBIP_cor) and skull (sBIP_ax, sBIP_cor) biparietal diameters and for transverse cerebellar diameter (TCD_ax, TCD_cor) using ICC, error rate, and paired Wilcoxon rank sum test. The *p*-value level for statistical significance was set at 0.05.

## Results

### Evaluation of Three-Dimensional Super-Resolution Reconstructions

A total of 26 cases were reconstructed based on the SR approach described above.

The estimated ordinal Gwet's AC between the three raters was 0.85 with a standard error of 0.06. According to Altman's benchmarking scale, this estimated coefficient was considered to be either *Good* or *Very Good* with a 0.99 probability.

As the inter-rater agreement was good, we considered the quality of a reconstruction as the averaged consensus between the three raters' assessment. On average, experts rated one case as bad, nine as average, and 16 as excellent (Figure 2B). No significant differences were found regarding the quality of the SR reconstruction and the gestational age (GA) ranges. The case rated as bad^1^ was discarded for further analysis; thus, 25 normal fetal brain MRI (mean GA: 27.1 ± 4.2 weeks, range: 18–34 weeks) were considered for the biometric analysis.

### Confidence of Measurements

On T2WS, some measurements were made with low confidence in three out of 25 fetuses (12%) by obs1, while obs2 reported a low level of confidence in 13 out of 25 fetuses (52%). Specifically, obs1 had low confidence in FOD in two fetuses and in axial measurements in DTC, sBIP, and bBIP in another fetus. In contrast, obs2 had low confidence mostly on the LCC (in 11 fetuses) and also in FOD and axial DTC (in two fetuses). On SR, low confidence measures were made only on the LCC in two fetuses by obs2. All the remaining measurements made on SR and T2WS were rated with high confidence. Overall, the level of confidence of obs2 was dependent on the dataset used, either T2WS or SR (*p* = 0.002), with higher confidence using SR. Conversely, no significant difference was found in the confidence level for obs1 (*p* = 0.23).

### Biometric Measurements Analysis

**Figure 4:**
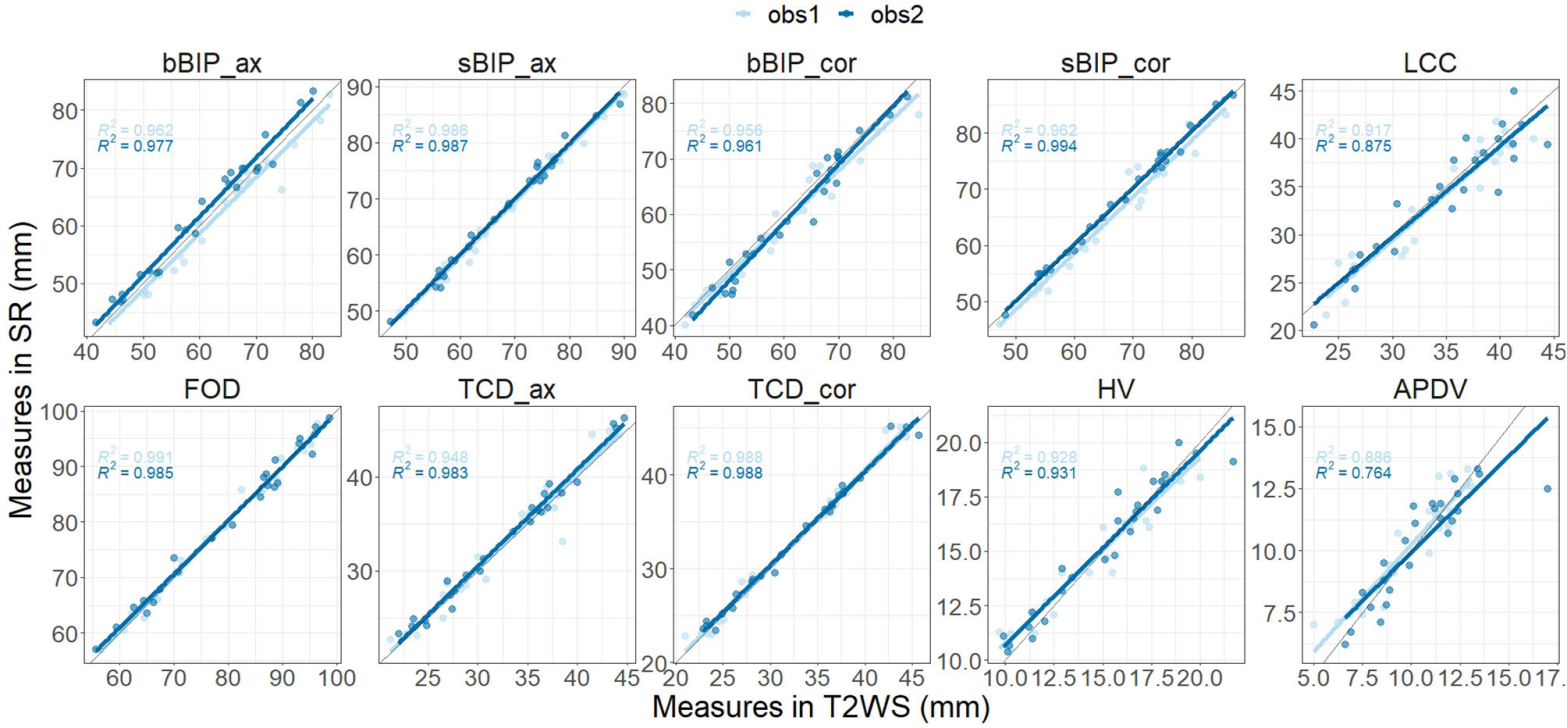
Inter-dataset [T2-weighted sequence (T2WS) vs. super-resolution (SR)] agreement for observer 1 and for observer 2 for each biometric measurement. For each biometric, measurements on SR (vertical y-axis) and on T2WS (horizontal x-axis) for each fetal brain MRI examination (point) for observer 1 (light blue) and observer 2 (dark blue). The solid gray line depicts perfect concordance.

Each observer–dataset pair showed a high correlation between right and left FOD (Spearman's rank correlation ρ = 0.975, *p* < 0.001) and no significant difference (*p* = 0.8). Hence, rFOD and lFOD were averaged. Agreement between T2WS and SR for each biometric parameter was good for both observers, with minimum Lin's CCC estimated to be 0.86. Both Lin's CCC and ICC were on average 0.997 for both raters (Figure 4; Table 2).

**Table 2:** Lin's concordance correlation coefficient values.

	**Obs1**	**Obs2**
bBIP_ax	0.972	0.977
sBIP_ax	0.992	0.994
bBIP_cor	0.969	0.971
sBIP_cor	0.973	0.996
LCC	0.951	0.932
FOD	0.996	0.992
TCD_ax	0.972	0.987
TCD_cor	0.992	0.992
HV	0.956	0.961
APDV	0.930	0.865

*Obs1, obs2, observer 1 and observer 2; LCC, length of the corpus callosum; APDV, anteroposterior diameter of the vermis; HV, height of the vermis; bBIP_cor, sBIP_cor, bBIP_ax, sBIP_ax, brain and skull biparietal diameter (coronal and axial); TCD_ax and TCD_cor, transverse cerebellar diameter (axial and coronal); FOD, fronto-occipital diameter*.

**Figure 5:**
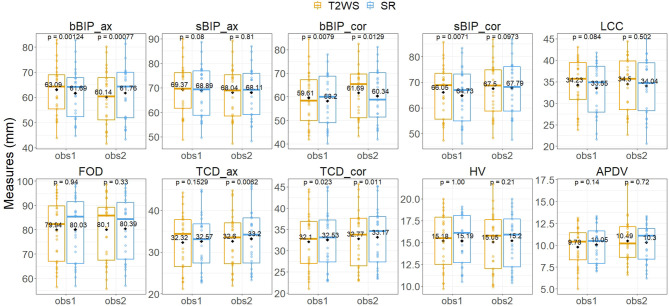
Inter-dataset intra-observer comparison for each biometric measurement using the paired Wilcoxon signed rank test. Vertical y-axis, value in mm of each biometric measurement for each fetal brain exam (point) for observer 1 (obs1) and observer 2 (obs2) (horizontal x-axis) for T2-weighted sequence (T2WS; orange) and super-resolution (SR; blue). Solid horizontal lines depict the median; diamonds depict the average of measurement in mm. See Figure 3 for measurement abbreviations. *p* < 0.05 is considered significant.

**Figure 6:**
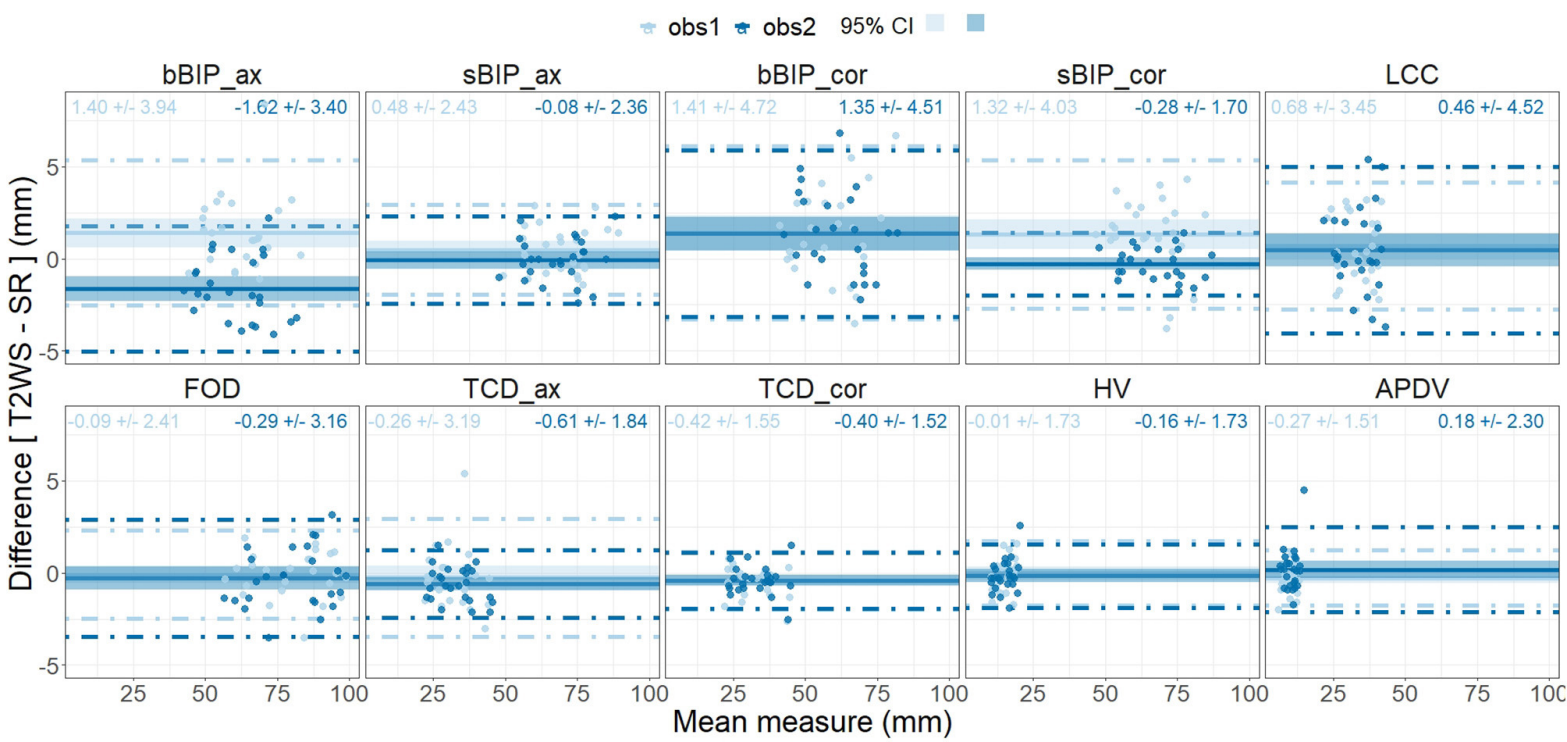
Bland–Altman plots of measurement differences between datasets [inter-dataset, T2-weighted sequence (T2WS) vs. super-resolution (SR)] for observer 1 and for observer 2 (intra-observer) for each biometric measurement. Each point corresponds in the x-axis to the mean measure between datasets for each observer (light blue for observer 1, dark blue for observer 2) and on the vertical y-axis to the difference between the two measurements T2WS/SR in mm. Horizontal solid line = mean of all measurement differences. Dashed lines = 95% limits of agreement, and shadow areas correspond to the 95% confidence interval (CI).

Inter-dataset (SR vs. T2WS) intra-observer (obs1 and obs2) comparisons (Figures 5, 6) showed statistical differences (*p* < 0.05) for brain axial and coronal BIP (bBIP_ax and bBIP_cor) for both observers, skull coronal BIP (sBIP_cor) for obs1, and axial and coronal TCD for obs2 (TCD_ax, TCD_cor). After correction for multiple comparisons, only axial brain BIP (bBIP_ax) remained significantly different for both observers, but differences were small (2.95 ± 1.73 mm) (Figure 6). Overall, the inter-dataset average error rate in our study was 3.3%. Additionally, Supplementary Table 3 shows a high intra-rater reliability for one observer on a sub-cohort of five fetuses.

**Figure 7:**
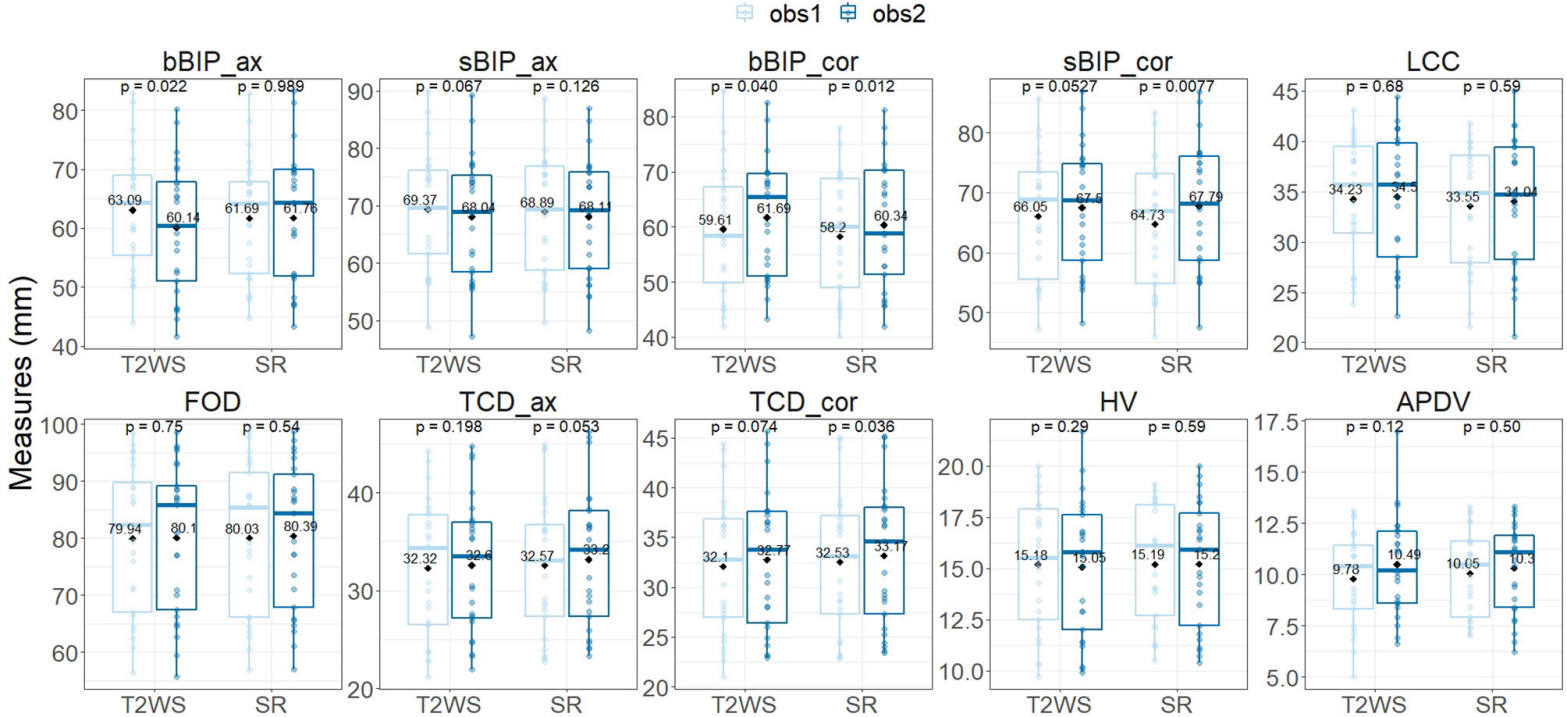
Inter-observer intra-dataset comparison for each biometric measurement using the paired Wilcoxon signed rank test. Vertical y-axis, measurement in mm for each biometric measurement for each fetal brain MRI exam (point) for T2-weighted sequence (T2WS) and super-resolution (SR) (horizontal x-axis) for observer 1 (light blue) and observer 2 (dark blue). Solid horizontal lines depict the median; diamonds depict the average of measurement in mm. See Figure 3 for measurement abbreviations. *p* < 0.05 is considered significant.

**Figure 8:**
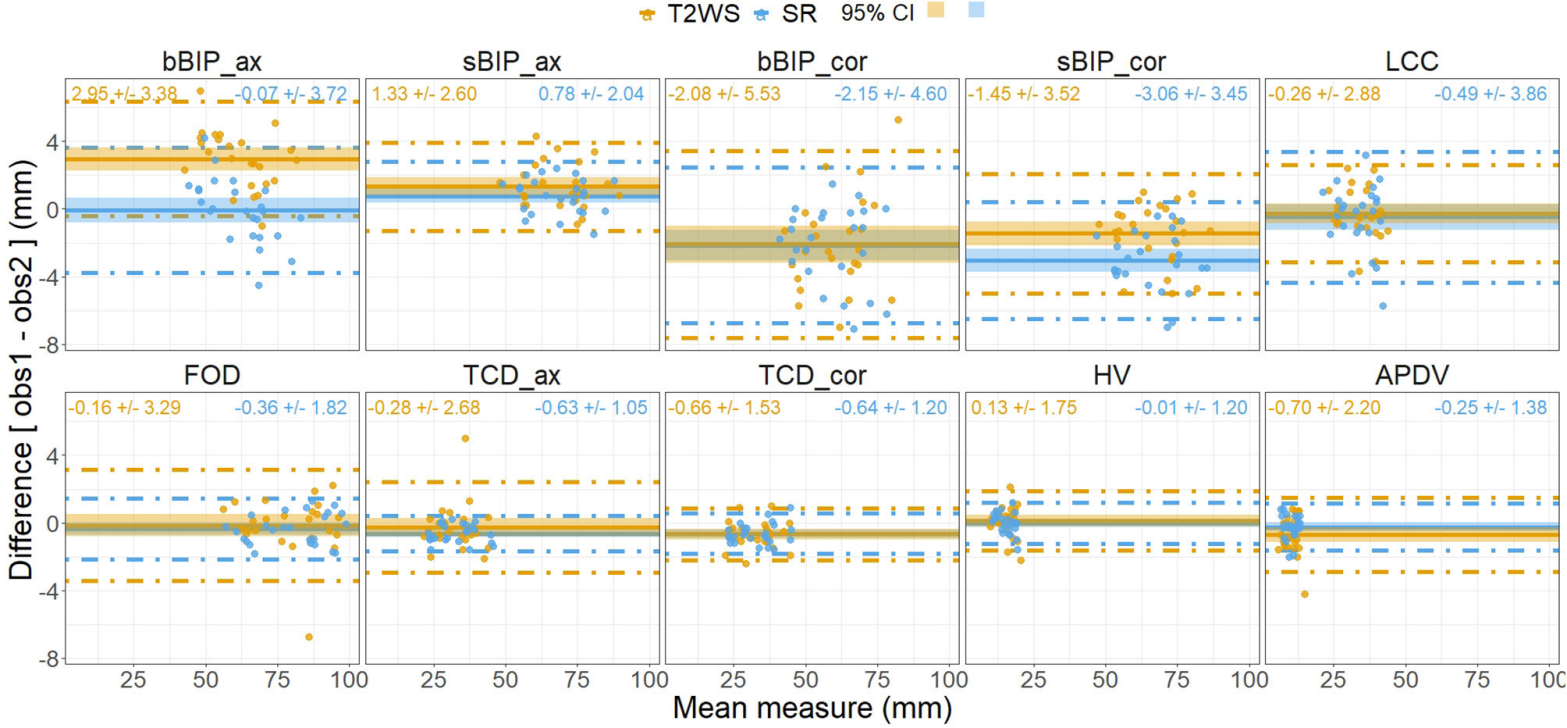
Bland–Altman plots of measurement differences between observers (observer 1 vs. observer 2) for each dataset [intra-dataset, T2-weighted sequence (T2WS) and super-resolution (SR)] for each biometric measurement. Each point corresponds in the x-axis to the mean measure between observers and in the vertical y-axis to the difference in mm between the two observers on T2WS (orange point) and SR (blue point). Horizontal solid line = mean of all measurements for T2WS (blue) and SR (red); dashed lines = 95% limits of agreement, and shadow areas correspond to the 95% confidence interval (CI).

Inter-observer intra-dataset comparisons showed no significant differences (*p* > 0.05), except for brain axial biparietal diameter (bBIP_ax) on T2WS and for brain and skull coronal BIP (bBIP_cor, sBIP_cor) and coronal transverse cerebellar diameter (TCD_cor) on SR. Overall, differences remained small (Figure 7) and independent of the measured size (Figure 8). After correction for multiple comparisons, none of them remained significant.

We computed the agreement between transverse and coronal measurements within each dataset and for both observers (ICC, percentage of error range, mean ± standard deviation of percentage error). Agreement was excellent without statistical differences (*p* > 0.05) for TCD on T2WS (ICC = 0.983, 0.3–16%, 2.9 ± 2.6%) and SR (ICC = 0.997, 0–1%, 1 ± 1%), for bBIP on T2WS (ICC = 0.951, 0.3–14%, 5.2 ± 3.5%) and SR (ICC = 0.970, 0.1–15%, 5 ± 3.8%), and for sBIP on T2WS (ICC = 0.972, 0–15%, 3.4 ± 3.2%) and SR (ICC = 0.972, 0–11%, 3.8 ± 3.4%). Complete results are presented in Supplementary Tables 1, 2.

## Discussion

### Summary of Contributions

Our study showed a sound agreement for biometric measurements performed on T2WS and on SR. Thus, a good-quality SR volume is as valuable as T2WS for fetal brain biometric assessment. Our results are in line with and complement a previous study that has also validated the use of SR for posterior fossa (29). Our SR biometric measures are also concordant with the normative charts of the fetal brain that have been recently published (28).

The intra-observer agreement in 25 fetuses (both Lin's CCC and ICC were on average 0.997 for both observers) is in line with previous work (28) that also compared T2WS and SR measurements and reported an ICC between 0.95 and 0.99. Furthermore, the error rate is also of the same range (3.3% in average in our study as compared to 0.2–2.4%). However, let us note though that in the work by Kyriakopoulou et al. (28), the comparison of T2WS vs. SR measurements was performed in a controlled sub-cohort of 10 fetuses specifically selected with symmetrical non-rotated images.

Intra-observer comparisons between T2WS and SR measurements showed significant differences only for axial brain BIP, though differences remained small (2.95 ± 1.73 mm) and acceptable in clinical practice, as age-specific reference intervals for this biometric parameter are larger (12). The discrepancies observed in our study for the axial brain BIP may be explained by a different plane used for SR and T2WS and the possibility offered by SR to change the windowing to more precisely identify anatomical landmarks. In contrast to US, there are no true standardized measurement criteria for the BIP on MRI; indeed, the chosen plane, axial or coronal, differs between authors. For instance, measurements of brain and skull BIP reference data were performed in the coronal plane through the temporal horns of the ventricles by some authors (4, 40), while it was made in the axial plane by others (28). We could question if the BIP obtained in the coronal plane really compares to the BIP obtained in the axial plane; however, our results still showed good agreement between transverse and coronal measurements for the BIP. A similar pattern was found for axial vs. coronal TCD, but with a shorter error interval for TCD in SR, similarly to another study (29) that also found superior concordance in SR rather than in T2WS measurement of TCD.

Our study also showed overall good inter-observer agreement for biometric measurements performed on T2WS and on SR, without any statistically significant difference after correction for multiple comparisons. This indicates that experience is not crucial for fetal brain biometric assessment and that SR can be used for biometry even by junior trainee radiologists without extensive expertise in fetal brain MRI. Indeed, obs2 did not report any fetal brain MRI before the study nor received special training. Obs1 showed obs2 how to perform the measurements on one subject and provided schematical guidelines similar to Figure 3. This is to be compared to recent work where the experience of the radiologist (number of previous fetal MRI exams reported) had an influence on the diagnostic error rate, with the less experienced radiologists having higher error rates of 11%, while experts had <3.8% (41).

In practice, the main additional value of SR is the possibility to reorient the planes in the standard anatomical planes using MPR. In our study, the junior radiologist had more confidence in identifying and measuring the corpus callosum on SR compared to T2WS. Indeed, it may be difficult to visualize the whole corpus callosum, particularly the rostrum, on conventional low-resolution 2D T2WS, and US is considered to have a better resolution in this case (42, 43). Our results suggest that SR makes it easier to identify the whole corpus callosum. The potential benefit of SR in this indication should be explored on larger series.

However, the 3D SR reconstruction also has some limitations. Obviously, the quality of SR depends on the quality of the native T2WSs, which are frequently affected by motion. Indeed, due to the reconstruction process itself, small or narrow structures (e.g., optic chiasm or corpus callosum) can appear blurred due to partial volume effects (27). Nevertheless, in our study, we obtained a good or excellent quality of SR images in 90% of cases, with a good visual rating concordance between three expert raters (one senior engineer and two senior radiologists). The fact that the concordance between T2WS and SR measurements was high indicates that the process of SR reconstruction does not distort the fetal brain anatomy (29). Our success ratio in SR reconstruction is slightly higher than the one previously reported at 3T (29), where 79% of 62 cases were successfully reconstructed.

SR reconstruction requires a minimum number of orthogonal series to ensure a good reconstruction quality (44, 45). In our study, six series were used on average (range of 3–11) per subject's reconstruction with an average computing time of 1 h. Previous works reported a similar number of series and processing time [from 4 to 15, in average eight series, 1–20 h in Gholipour et al. (27); and eight series and 40 min in Kyriakopoulou et al. (28)]. The processing time, the need for user interaction for the series selection or the refinement of brain masks, and the lack of integration into the clinical environment preclude the use of SR volumes for fetal brain exploration in daily practice. However, current developments aim at automatizing and accelerating SR reconstruction (24, 25, 34) to facilitate its integration as a supporting tool in clinical routine and consequently also to be adopted by non-engineer users.

The clinical adoption of SR-reconstructed fetal MRI is thus at its earliest stage (46–49). In our center, SR-reconstructed MRI is used only for certain cases, in particular to confirm or reject suspected cortical abnormalities on 2D T2WS. In our opinion, for now, SR could be complementary to but cannot replace low-resolution 2D T2W planes for brain parenchyma analysis, as voxel intensities in SR volumes have undergone many changes. Indeed, beyond the promising value of SR fetal brain biometry, the intensity contrast of SR images still has to be evaluated for its diagnostic value in comparison to native T2WS and eventually improved. Let us recall that voxel intensities in the reconstructed SR volume are computed from the original voxel values within the multiple series throughout many image processing steps; therefore, they are not directly generated from the MRI scanner, and their interpretation as pathological features has to be done with caution.

### Strengths and Limitations

Our study is the first to compare both supratentorial and infratentorial measurements with SR, while only posterior fossa measurements were made in Pier et al. (29); this latter study included only normal volunteers, while our cases were taken from clinical workflow. We also compared SR measurements between a junior and a senior radiologist to evaluate the influence of experience on biometric results. We have shown that even a radiologist without expertise in fetal brain MRI can perform accurate fetal brain biometry—thanks to SR. In contrast, multiple raters who received extensive training developed normative charts for 2D T2WS and 3D SR fetal brain measurements (28). In their study, the agreement analysis was performed between measurements on native T2WS and SR in a sub-cohort of only 10 fetuses, with the same rater, and on selected symmetrical non-rotated images. Our study on 25 fetuses comparing two raters provides additional insight into the value of SR techniques for biometry of fetal brain MRI.

A limitation of our study is the small-size cohort, and so the few subjects available per gestational week. For this reason, we cannot draw conclusions about gestational age or sex influence on measurement quality. Finally, the reproducibility of our measurements were tested only with one observer on a sub-cohort of five fetuses.

## Conclusion

This study demonstrates that SR is a valid, reliable, and simple method for biometric measurements. SR measurements are concordant with T2WS measurements, even when conducted by non-expert radiologists. Some biometric measurements like the biparietal diameter show small statistically significant differences, which can be explained by poorly defined and standardized measurement criteria. As soon as full automatization of SR is available in the clinical environment, the use of 3D SR could initially complement conventional T2WSs by faster providing the reference planes and further facilitating biometric measurements. Afterward, SR could provide a new standard of measurements in real orthogonal planes more than mimicking ill-defined or incorrect 2D planes. Future studies on pathological cases will enable the evaluation of other potential benefits of SR in clinical practice.

## Data Availability Statement

The data analyzed in this study is subject to the following licenses/restrictions: the ethical approval for the use of these data did not include public release. Requests to access these datasets should be directed to Meritxell Bach Cuadra, meritxell.bachcuadra@unil.ch.

## Ethics Statement

The studies involving human participants were reviewed and approved by Commission cantonale (VD) d'éthique de la recherche sur l'être humain (CER-VD). Written informed consent for participation was not required for this study in accordance with the national legislation and the institutional requirements.

## Author Contributions

MKh, PDu, VD, MB, and MKo contributed to the conception and design of the study and wrote sections of the manuscript and the first draft of the manuscript. PH, PM, RM, and VD acquired the images and evaluated the subjects used in this study. MKh and MKo performed biometric measurements. PDu and VD performed statistical analysis. MB performed the image reconstruction. VD, MB, and MKo evaluated the images. PDe, ST, and MB contributed to the software development used in this study. HL, HK, TY, and J-PT contributed to the analysis and interpretation of the results. All authors contributed to manuscript revision and read and approved the submitted version.

## Conflict of Interest

The authors declare that the research was conducted in the absence of any commercial or financial relationships that could be construed as a potential conflict of interest.

## Publisher's Note

All claims expressed in this article are solely those of the authors and do not necessarily represent those of their affiliated organizations, or those of the publisher, the editors and the reviewers. Any product that may be evaluated in this article, or claim that may be made by its manufacturer, is not guaranteed or endorsed by the publisher.

## Footnotes

^1^Bad SR quality was primarily due to only four series were available and brain was cut in two of them.
