# Supplementary material for: Fetal Brain Biometric Measurements on 3D Super-Resolution Reconstructed T2-Weighted MRI: An Intra- and Inter-observer Agreement Study
Source: Front Pediatr. 2021 Aug 10;9:639746. doi: 10.3389/fped.2021.639746 (PMC8383736; doi:10.3389/fped.2021.639746)
Supplement: Supplementary file 1 [file Table_1.DOCX]

Supplementary Material


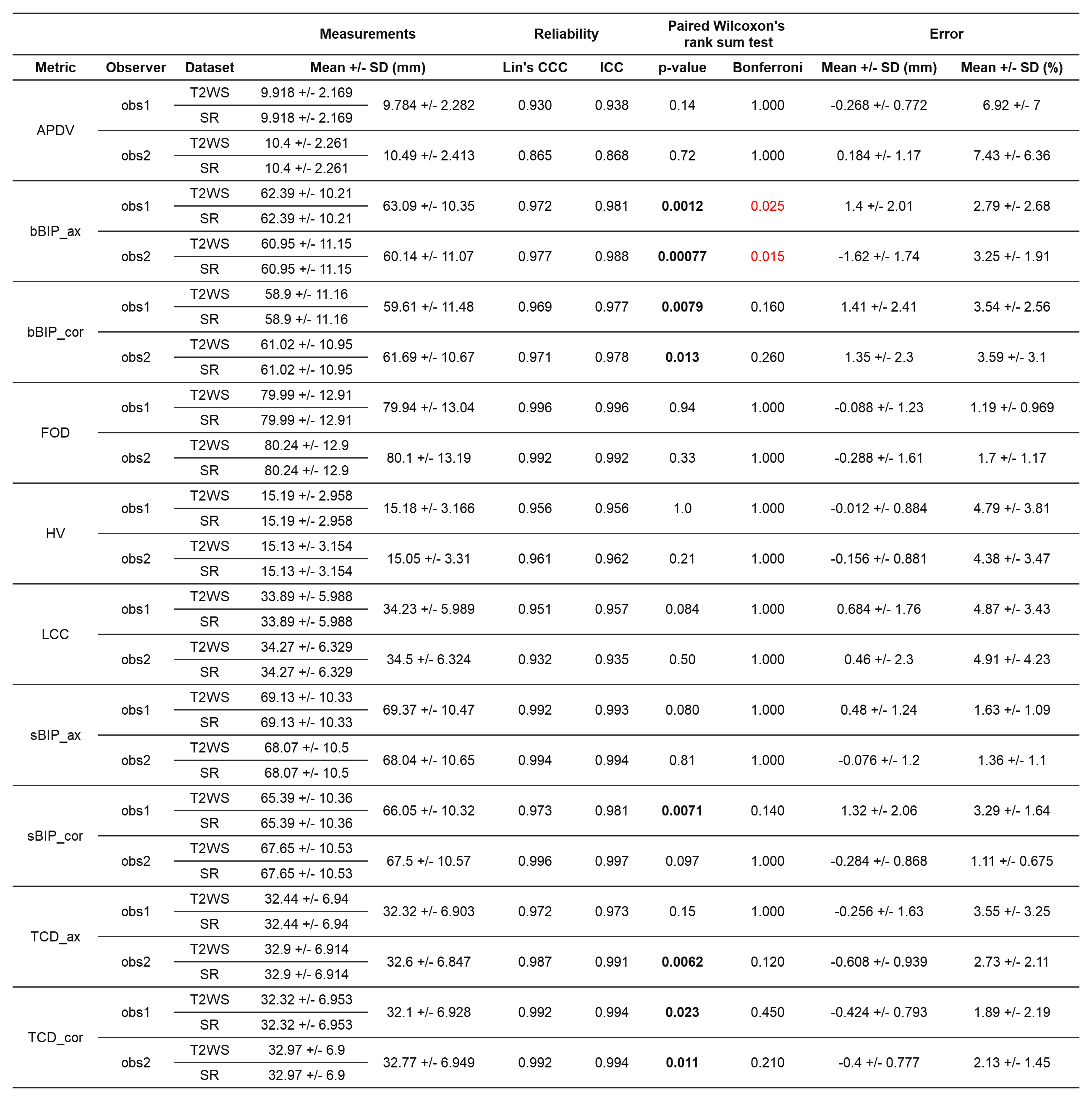


**Supplementary Table 1.** Summary table of inter-dataset intra-observer analysis for each biometric measurements: mean and standard deviation, agreement analysis (Lin’s concordance Correlation Coefficient and Intraclass Correlation Coefficient), comparison using paired Wilcoxon’s rank sum test (without correction (bold: significant p values) and with Bonferroni multiple comparisons correction (red: significant p values after correction)) and measurement error (in both mm and in percentage).


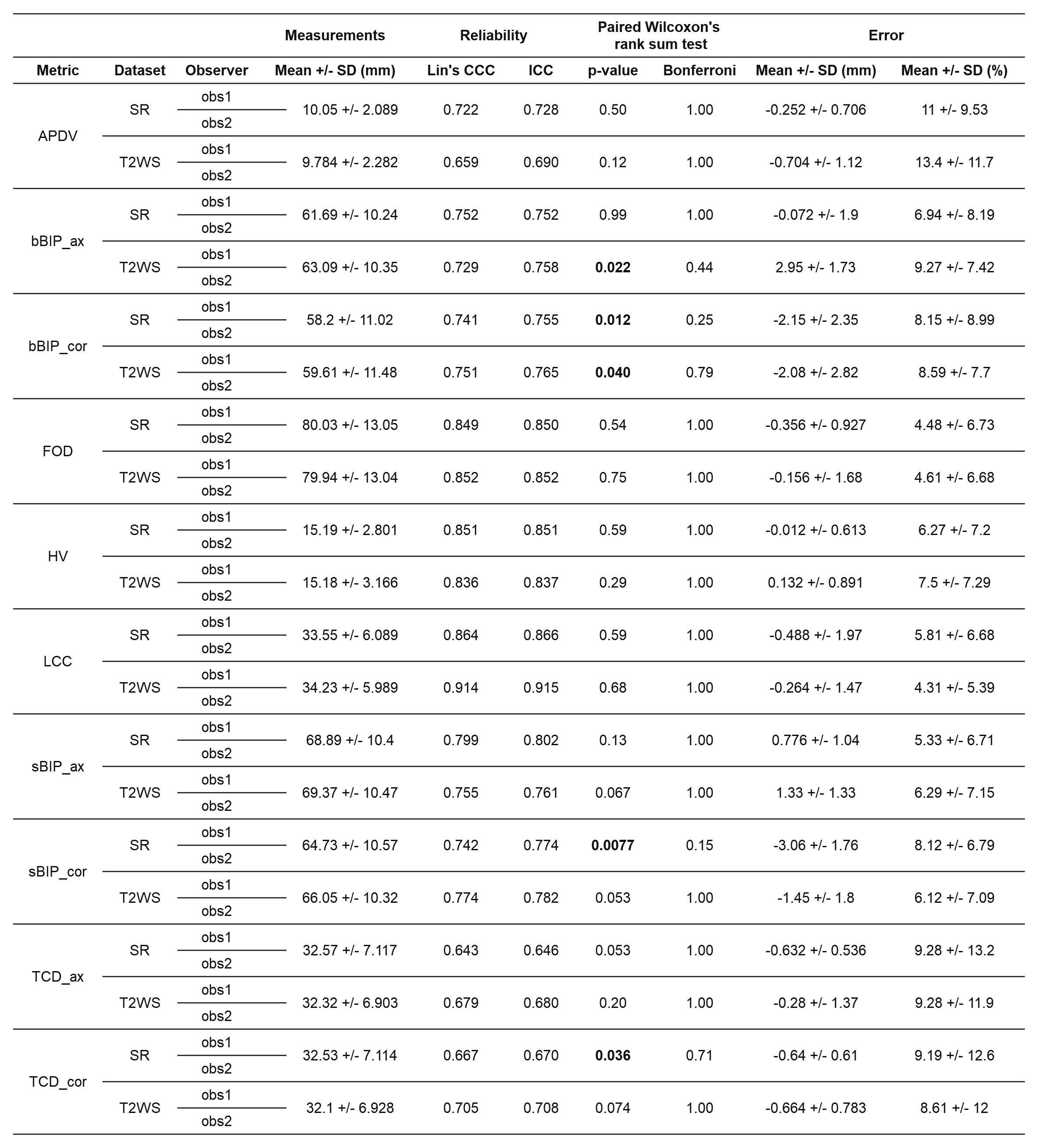
 **Supplementary Table 2.** Summary table of inter-observer intra-dataset analysis for each biometric measurements: mean and standard deviation, agreement analysis (Lin’s concordance Correlation Coefficient and Intraclass Correlation Coefficient), comparison using paired Wilcoxon’s rank sum test (without correction (bold: significant p values) and with Bonferroni multiple comparisons correction (red: significant p values after correction)) and measurement error (in both mm and in percentage).

**Supplementary Table 3. Intra-rater analysis.** To ensure the reliability of the measures, we have performed a complementary intra-rater variability analysis with obs1. This additional analysis was performed in a sub-cohort of five fetuses (gestational ages: 18, 22, 26, 30 and 34 weeks). For each subject, obs1 repeated 3 times the measurement of all the biometrics (measurements 2 and 3 were done one week apart, approximatively a year after measurement 1), on both T2WS and SR datasets. We present in this table the intra-class correlation (ICC) of the repeated measures by obs1 for each biometric and dataset. Overall, the intra-rater reliability is excellent with ICC = 0.97. Similarly for each dataset, ICC=0.97 and 0.97 respectively for T2WS and SR dataset. For SR, this is in line with (Kyriakopoulou et. *al*, 2017) that presents an ICC of 0.99.

|  | LCC | HV | APDV | DTC_cor | DTC_ax | sBIP_cor | sBIP_ax | bBIP_cor | bBIP_ax | DFO | **ALL** |
| --- | --- | --- | --- | --- | --- | --- | --- | --- | --- | --- | --- |
| T2WS | 0.83 | 0.87 | 0.77 | 0.97 | 0.97 | 0.87 | 0.81 | 0.91 | 0.88 | 0.88 | **0.97** |
| SR | 0.85 | 0.88 | 0.85 | 0.96 | 0.96 | 0.85 | 0.85 | 0.90 | 0.86 | 0.88 | **0.97** |
| Overall | 0.84 | 0.87 | 0.79 | 0.96 | 0.96 | 0.86 | 0.83 | 0.90 | 0.87 | 0.88 | **0.97** |
